# Deep venous thrombosis and abortion: an unusual clinical manifestation of severe form of pectus excavatum

**DOI:** 10.1007/s11748-020-01583-0

**Published:** 2021-01-27

**Authors:** M. Bianco, S. Mantovani, F. G. D’Agostino, M. Bassi, D. Amore, S. Cagnetti, E. Mottola, J. Vannucci, F. Venuta, M. Anile

**Affiliations:** Department of Thoracic Surgery, “Sapienza” University of Rome, Viale del Policlinico 155, 00161 Rome, Italy

**Keywords:** Pectus excavatum, Thrombosis, Sternochondroplasty

## Abstract

Pectus excavatum is a chest wall malformation with a strong psychological and aesthetic impact. Rarely, pectus excavatum patients can show respiratory or cardiac symptoms occurring mainly during physical exertion. We report a case of a 34-year-old pregnant woman with a severe degree of pectus excavatum who developed serious cardiovascular disease resulting in spontaneous twin abortion at the twenty-first week of gestation. Cardiovascular disease was resolved after open surgical correction of pectus excavatum. This case shows how a tardive diagnosis and a delayed surgical approach for pectus excavatum can lead to severe consequences.

## Introduction

Pectus Excavatum is the most common deformity of the chest wall [1]. Generally, pectus excavatum has only a strong psychological and aesthetic impact, especially in youth and adolescence, showing alterations of their emotional and behavioral sphere, manifesting a real discomfort with their body and aesthetic appearance. In rare cases, pectus excavatum can determine the appearance of cardio-respiratory symptoms occurring mainly during physical activity such as arrhythmias, dyspnea, reduced cardiopulmonary fitness, compromised exercise capacity, and fatigue [2]. In this article, we describe a case of a 34-year pregnant woman with a severe degree of pectus excavatum determining heart compression and resulting in a spontaneous twin abortion at the twenty-first week of gestation due to a venous thrombosis.

## Case

**Fig. 1:**
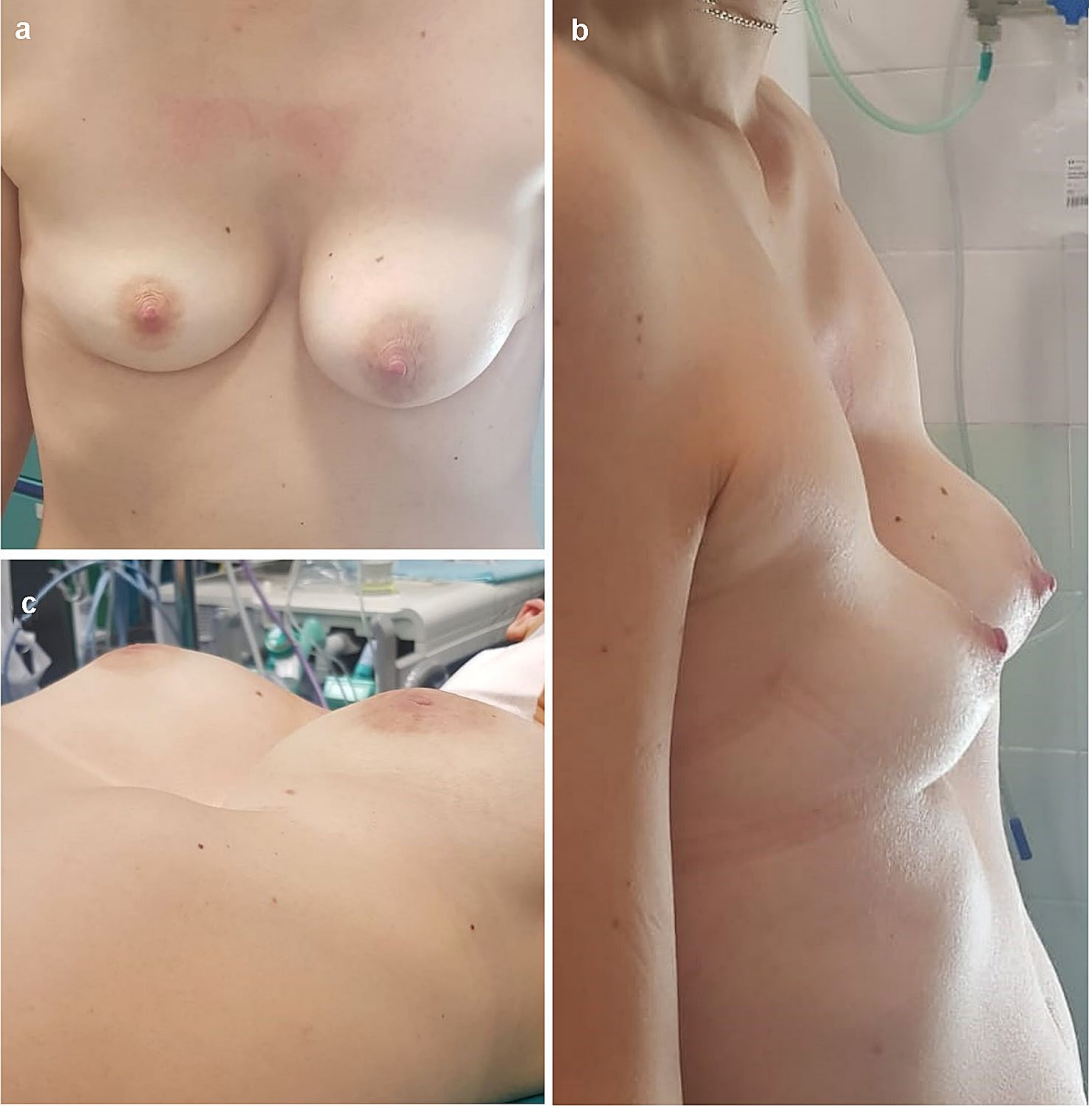
Preoperative view of pectus excavatum. **a** frontal plane **b** sagittal plane **c** transverse plane

**Fig. 2:**
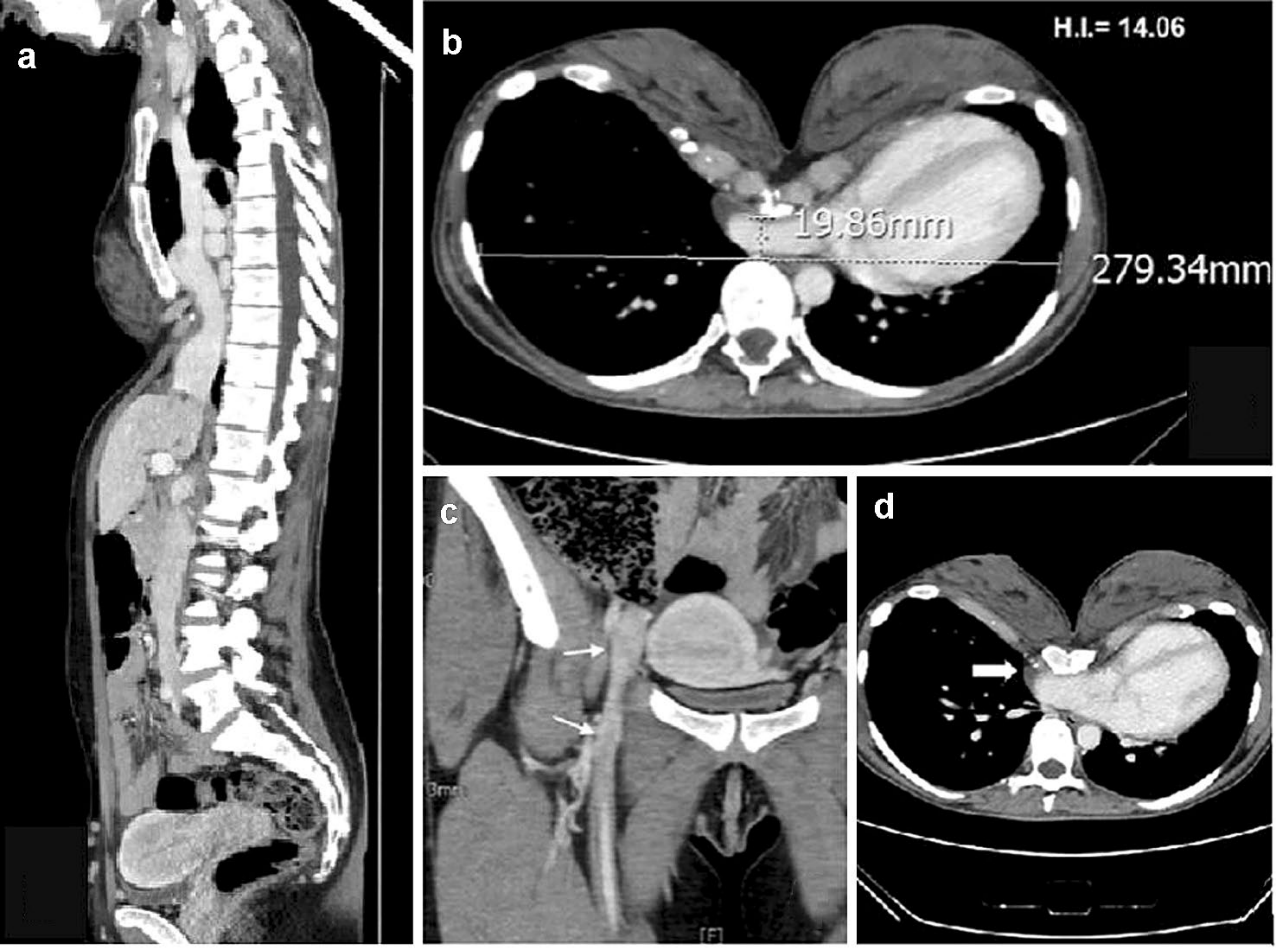
**a** Preoperative sagittal CT scan showing the deep depression of the sternal corpus. **b** Preoperative axial CT scan with Haller Index. **c** Preoperative coronal CT scan showing thrombosis in the deep femoral system. **d** Preoperative coronal CT scan showing thrombosis in the inferior vena cava

In December 2019, a 34-year-old woman was referred to our Thoracic Surgery Unit with a severe pectus excavatum (Fig. 1a–c). According to Chin classification [3], computed tomography (CT) of the chest revealed a type I symmetric PE involving from 4th rib to 7th rib with a Haller index of 14.06, compression of inferior vena cava (IVC) and right atrium with thrombotic processes affecting the inferior vena cava up to the superficial and deep femoral system (Fig. 2a–d).

The patient did not refer any psychological impairment during her life, but only exertional dyspnea that has been always attributed to asthma, even if not under pharmacological treatment. On March 2019, she experienced a spontaneous twin abortion at the 21st week of gestation, probably due to deep vein thrombosis; no autoimmune disorders (negative anticardiolipin and homocysteine antibodies) or coagulopathies were detected. Therefore, she started therapy with direct oral anticoagulants without obtaining a complete resolution of the thrombotic process, as demonstrated by the echo-color Doppler examination of the lower limbs performed after 6 months. Preoperative transthoracic echocardiogram showed normal ejection fraction (EF: 68%) and the presence of mild mitral and pulmonary valve insufficiency, with systolic retroflexion of the anterior flap of the mitral; in subcostal view, the inferior vena cava flow appears reduced and turbulent.

**Fig. 3:**
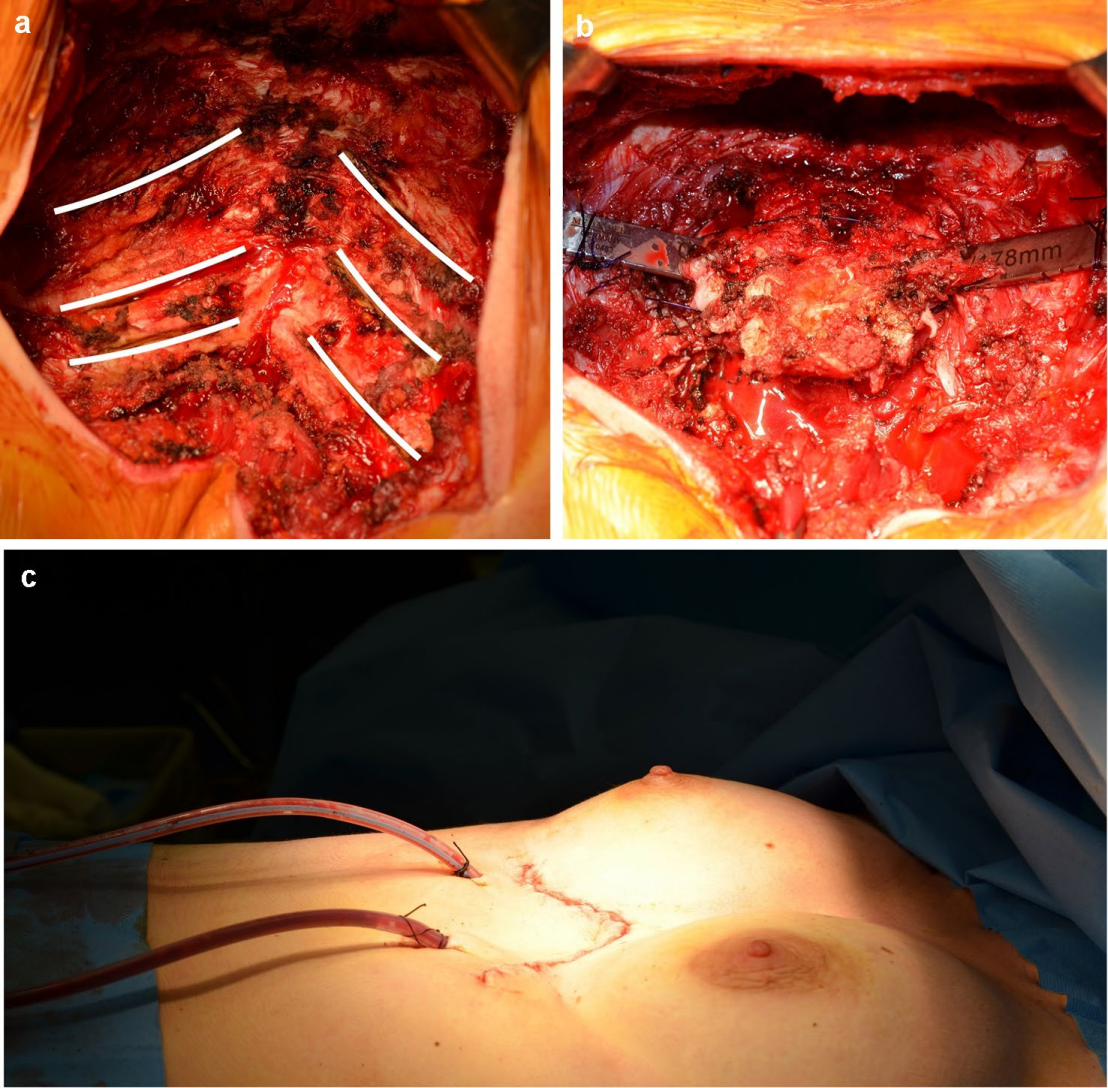
Intraoperative view. **a** Bilateral removal of the costal cartilages (white lines). **b** Steel bar placed under the sternum. **c** Submammary incision at the end of surgery

**Fig. 4:**
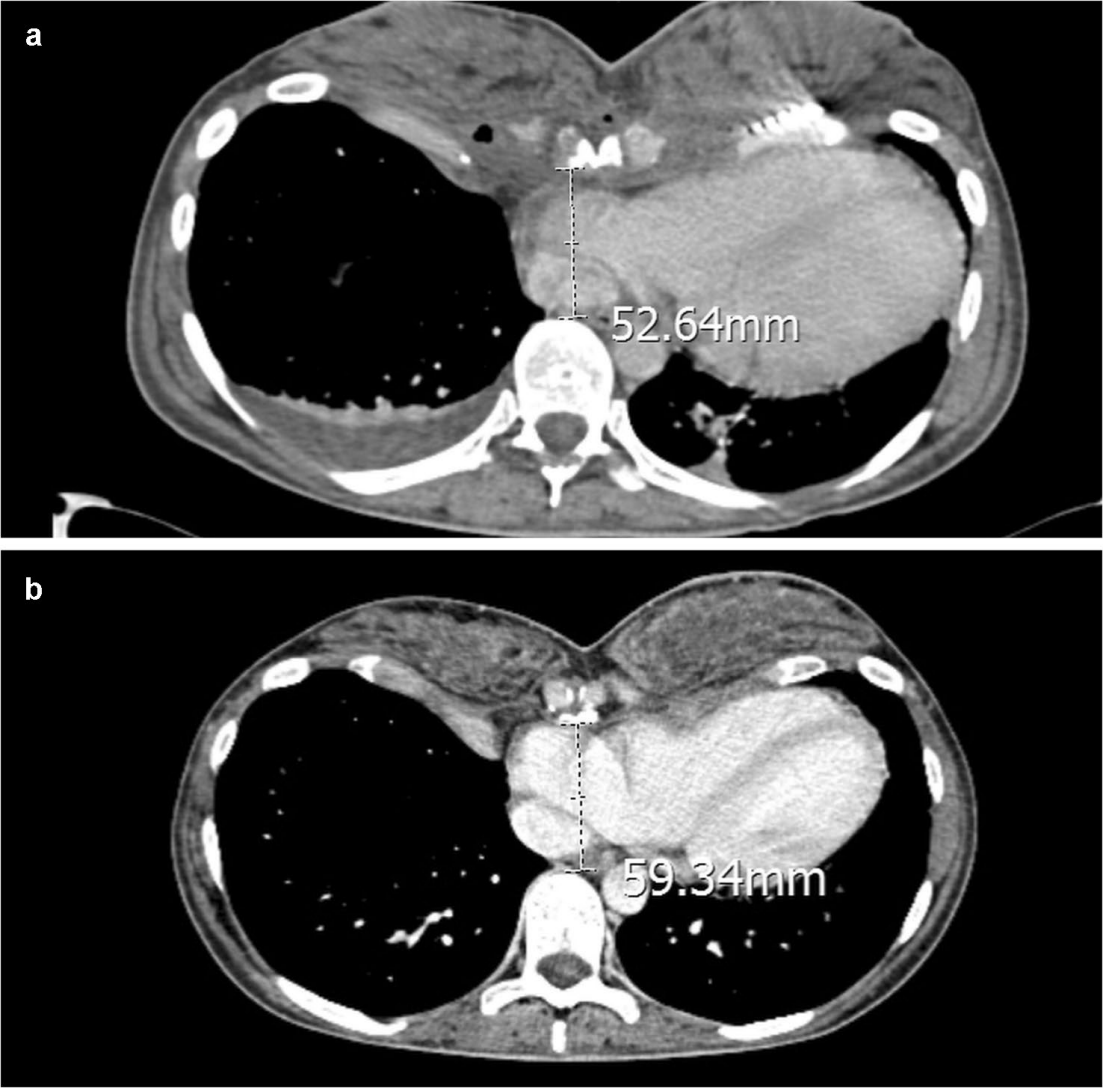
**a** Postoperative axial CT scan at 3rd day showing improvement of antero-posterior diameter. **b** Postoperative axial CT scan at 6 months showing further improvement of antero-posterior diameter

Due to the severity of the pectus excavatum, the mini-invasive approach was excluded [4] and she underwent sternochondroplasty by Ravitch’s approach through a submammary incision; after the bilateral removal of the costal cartilages from IV to VII ribs, we performed a transverse osteotomy in the sternal corpus and placed a steel bar below the sternum to elevate it (Fig. 3a–c). Operation time was 110 min. After 3 days, postoperative CT showed reduction of compressive effects on the heart, in particular on the right atrium and inferior vena cava, both regularly opacified by the contrast. The antero-posterior diameter of the chest, measured at the deepest level, increased from 19.86 mm (preoperative) to 52.64 mm (postoperative) with a Haller index of 5.08. The patient was discharged on the fifth postoperative day; a 6-month chest CT showed a further improvement both of Haller index (4.73) and the antero-posterior diameter (59.34 mm) (Fig. 4a, b); the thrombosis was no longer evident at the level of the femoral vein and the patient has discontinued the anticoagulant therapy. Even spirometry performed after surgery showed no signs of obstructive pulmonary disease, demonstrating how the diagnosis of asthmatic disease was inexact. Postoperative echocardiography showed a reduction of mitral and pulmonary valve insufficiency with improvement of cardiac function and the inferior vena cava flow was clearly visualized.

## Discussion

Aesthetic discomfort is the main indication for pectus excavatum correction. The pectus excavatum-related complications are known until decades and clinical presentation can be various. Hemodynamic complications, arrhythmias, decrease of pulmonary function and dyspnea are rare, but possible and they can be considered in decision-making process [4]. In our clinical case, however, the patient had never experienced cardiovascular disorders before pregnancy and she never had shown aesthetic or psychological impairment due to the congenital malformation. The mechanism underlying our clinical situation is probably due to physiological modifications occurring during twin gestation and pectus excavatum: the overload on the compressed heart modifies the blood flow balance, determining the IVC thrombosis. The prothrombotic state, induced by pregnancy, typically results as a deep iliac and femoral system thrombosis, and pulmonary embolism is a common and dangerous complication; however, the right atrium and inferior vena cava thrombosis is a rare condition and in this case the pectus excavatum works as a mechanical risk factor. We suppose, on the basis of the preoperative imaging and her medical history, that IVC thrombosis is strongly related with the abortion, but the real mechanism is unclear. At the time of spontaneous abortion, the deep iliac thrombosis was demonstrated by echo-color Doppler and the echocardiography shows indirect sign of IVC thrombosis. The patient was referred to our department after 6 months of anticoagulant therapy and CT scan shows minimal residual thrombosis at atrial–IVC junction; for these reasons, we can speculate, but not demonstrate by imaging the direct correlation between thrombosis and abortion.

For the first time, we present the case of IVC compression and thrombosis due to pectus excavatum in pregnant woman who presents abortion, and the relationship is not descript before in literature. White et al. [5] described the case of pectus excavatum with compression of IVC and recurrent syncope in 22-year-old woman; they proposed that the compression of the sternum on right atrium and the reduction in upright cardiac output caused the disorder. Lannucci et al. in 2015 described the case of lower extremity edema in a child due to pectus excavatum; after 3 months of surgical correction, the patient presents complete resolution of her edema and leg pain [6].

Although the mini-invasive approach is actually the first surgical choice [7, 8], in this case, the severity of malformation and the impressive heart compression have suggested a traditional surgical approach. We obtained a progressive increase of the antero-posterior diameter of the chest allowing the decompression of the caval–atrial junction, a correct venous return to the heart and consequently to the systemic circulation with an improvement in the overall cardio-circulatory performance. The patient has no more exertional dyspnea and does not show edema in the lower limbs with an improvement of quality of life.

In conclusion, this particular case shows that a delayed diagnosis of a severe form of pectus excavatum can lead to sudden symptomatic and unfortunately dramatic manifestations. Corrective surgery has a high success rate, but the timing and surgical approach should be determined on case-by-case.
